# An Ultrasound Imaging-Guided Robotic HIFU Ablation Experimental System and Accuracy Evaluations

**DOI:** 10.1155/2017/5868695

**Published:** 2017-04-13

**Authors:** Chih Yu An, Jia Hao Syu, Ching Shiow Tseng, Chih-Ju Chang

**Affiliations:** ^1^Department of Mechanical Engineering, National Central University, Taoyuan County 32001, Taiwan; ^2^Graduate Institute of Biomedical Engineering, National Central University, Taoyuan County 32001, Taiwan; ^3^Department of Neurosurgery, Cathay General Hospital, Taipei City 10630, Taiwan; ^4^Department of Medicine, School of Medicine, Fu Jen Catholic University, New Taipei City 24205, Taiwan

## Abstract

In recent years, noninvasive thermal treatment by using high-intensity focused ultrasound (HIFU) has high potential in tumor treatment. The goal of this research is to develop an ultrasound imaging-guided robotic HIFU ablation system for tumor treatment. The system integrates the technologies of ultrasound image-assisted guidance, robotic positioning control, and HIFU treatment planning. With the assistance of ultrasound image guidance technology, the tumor size and location can be determined from ultrasound images as well as the robotic arm can be controlled to position the HIFU transducer to focus on the target tumor. After the development of the system, several experiments were conducted to measure the positioning accuracy of this system. The results show that the average positioning error is 1.01 mm with a standard deviation 0.34, and HIFU ablation accuracy is 1.32 mm with a standard deviation 0.58, which means this system is confirmed with its possibility and accuracy.

## 1. Introduction

Cancer is a major public health problem all over the world. According to statistics, common cancers in both sexes happened in the lungs, breasts, colorectum, prostate, stomach, liver, and cervix uteri [1]. Take liver tumor as an example. Early diagnosis and treatment of liver disease are very important measures to avoid worsening. Except biochemical tests such as GOT/GOP or *α*-globulin, ultrasound scanning is usually adopted for first-line screening and diagnosis. If the disease needs further treatment, tissue biopsy, percutaneous ethanol injection, or RF burning will be usually done under ultrasound guidance. For serious cases, open or minimally invasive liver dissection treatment will be necessary. However, all of them are invasive treatments.

Recently, a noninvasive high-intensity focused ultrasound (HIFU) thermal treatment has demonstrated high potential on tumor treatment. The physical principle of this interventional approach is to apply focused ultrasound waves to the tumor tissue such that the heating of the tissue causes its necrosis [2]. The concepts of noninvasive surgery using HIFU had been proposed by Lynn et al. in 1942 [3]. Later in 1960, W. J. Fry and F. J. Fry used HIFU to treat the patients who were suffering from various neurological disorders. Their study shows that the HIFU beams can be used to produce changes in practically any desired brain structure. And the changes can be induced without adversely affecting the intervening brain structure and without interrupting the vascular system even within the site in which irreversible or permanent changes in the neural components are produced [4]. During the past decade, HIFU therapy has been successfully delivered clinically to many lesions, including the prostate tumor [5, 6], uterine fibroids [7, 8], and liver tumor [9].

During the HIFU treatment, we need the information of the target tumor in order to calculate the size and location of the ablation zone. Kim et al. used magnetic resonance imaging (MRI) to guide HIFU ablation on 33 uterine fibroid patients. Targeting accuracy values (displacements in absolute values) were 0.9 ± 0.7 mm, 1.2 ± 0.9 mm, and 2.8 ± 2.2 mm in LR, CC, and AP directions, respectively. Of 527 sonications, 99.8% (526 of 527) were within acceptance ranges [10]. Holbrook et al. focused on the distance error problem of liver tumor which is caused by respiration. MRI is chosen by them to build in their system to guide HIFU focal point and track the moving phantom [11]. Except MRI, ultrasound imaging (USI) is another choice for researches. Sakuma et al. developed a HIFU treatment system for minimally invasive liver surgery which integrated a three-dimensional USI system in 2002. Their study shows that navigation errors were within 3 mm [12]. Later in 2015, Peng et al. investigated the value of microbubble contrast-enhanced ultrasound (CEUS) in evaluating the treatment response of uterine fibroids to HIFU ablation and also compared the value with MRI. The result shows that CEUS has the ability to show the size of fibroids and the nonperfused areas of the fibroid clearly. And the results from CEUS correlated well with the results obtained from MRI [13]. From those literature reviews, we know that both MRI and USI can be chosen to guide HIFU ablation. MRI has its advantages in offering better images as well as temperature monitoring in the target zone [14]. However, the treatment time of MRI-guided HIFU procedure is longer than that of USI-guided HIFU. The cost of MRI-guided HIFU is also much more than that of USI-guided HIFU [15]. In addition, USI can apply the real-time images during the HIFU treatment. It is an advantage for the HIFU treatment.

Additionally, since the target tumor we faced is much larger than the size of the HIFU focal point, the treatment of the entire volume of tumor is not suitable for handheld HIFU transducer. Besides, HIFU treatment needs 0.5~5 s to ablate a single point. The stability and the positioning accuracy of the HIFU focal point should take a serious concern. Chauhan and ter Haar developed a HIFU treatment system named FUSBOT which combined with the robotic arm to achieve the stability and the positioning accuracy of HIFU focal point. According to their study, the navigation errors were within 0.5 mm [16, 17]. Masamune et al. developed another HIFU positioning robot treatment system which integrated the HIFU transducer, ultrasound probe, and a robotic arm (which has 4 degrees of freedom) for fetal sacrococcygeal teratoma treatment. The positioning errors of their robotic arm in X, Y, and Z directions were −0.2 ± 0.3 mm, −0.1 ± 0.1 mm, and −0.0 ± 0.1 mm, respectively [18]. As shown above, most of the studies combined their system with a robotic arm (with different DOF or mechanism) to facilitate the stability and the accuracy of HIFU treatment system.

Eventually, it is quite difficult to assess the quality of this noninvasive therapy, and there is a dire need for a high-accuracy system supporting in planning, conducting, and monitoring such treatment. Therefore, this research aimed to study and develop an ultrasound imaging-guided robotic HIFU ablation system for tumor treatment. Instead of building a huge, solid, and expensive system, our HIFU ablation system combined with the existing ultrasound imaging equipment to achieve HIFU ablation function. The previous studies of this system were already revealed in the conference of Biomedical Electronics and Devices in 2015 [19].

## 2. Material and Methods

### 2.1. The Structure of the Ultrasound Imaging-Guided Robotic HIFU System

**Figure 1:**
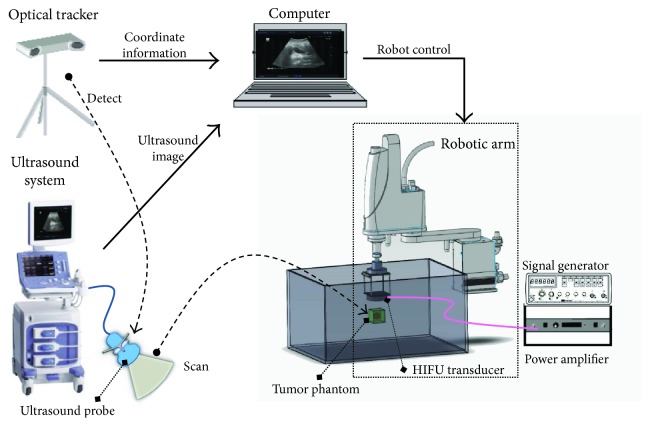
The ultrasound imaging-guided robotic HIFU system.

As shown in Figure 1, the ultrasound imaging-guided robotic HIFU system integrates the ultrasound imaging system (ALOKA, Prosound Alpha 6), the HIFU ablation system (Sonic H-106 probe with Instek, GFG-8255 signal generator and AR, and 150A100B power amplifier), the robotic arm (YAMAHA, YK400XG), the optical tracker (Northern Digital, Polaris Spectra), and a notebook (Dell, M4500) into this system.

**Figure 2:**
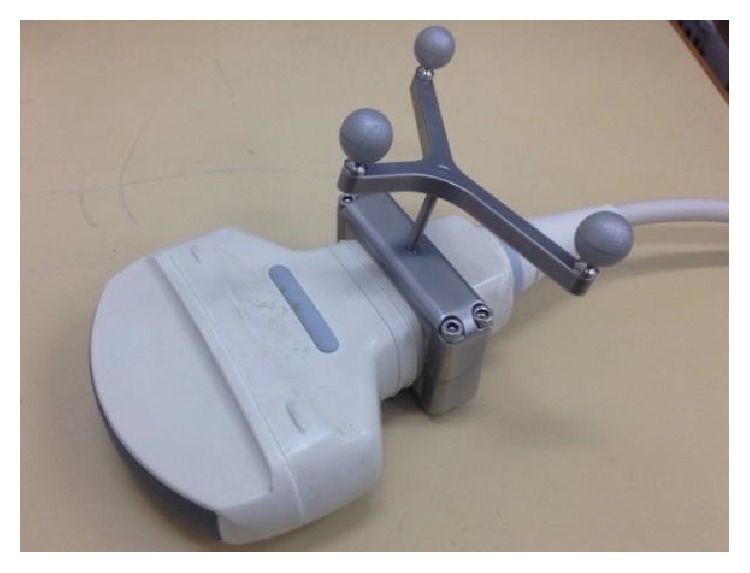
A DRF set on an ultrasound probe.

The ultrasound probe scans the tumor phantom to obtain the location of the tumor. The movement of the ultrasound probe is controlled by the motor-driven linear slide and detected by the optical tracker through the DRF (dynamic reference frame, a tool with three IR-reflective marker spheres, as shown in Figure 2), which is a reference coordinate frame tracked by the optical tracker. Through coordination transformation described below, the position of the tumor phantom relative to the ultrasound image frame can be transferred and represented by the robot frame. The robotic arm is thus able to bring the focal point of the HIFU transducer to aim at the tumor phantom. The signal generator and power amplifier are used to enable the HIFU transducer to generate high-intensity sound power for thermal therapy.

**Figure 3:**
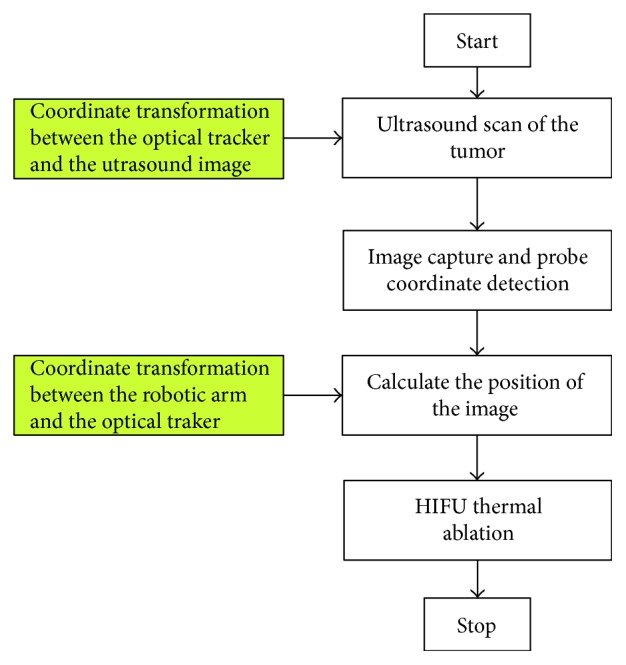
The working procedures of the ultrasound imaging-guided robotic HIFU system.

The blocks in the right column of Figure 3 show the working procedures of the ultrasound imaging-guided robotic HIFU system. Green blocks in the left column represent the preliminary works before starting the system. They are also the key points of this study which will be described clearly in the next sections.

### 2.2. Coordinate Transformation between the Optical Tracker and the Ultrasound Image

**Figure 4:**
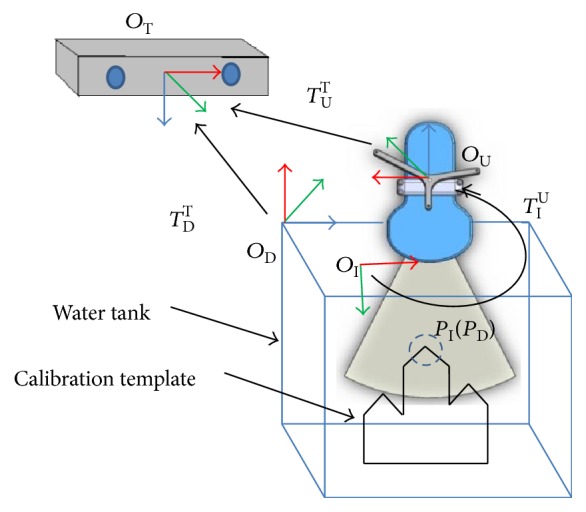
The coordinate transformation between the optical tracker and the ultrasound image.

Figure 4 illustrates our method for determining the coordinate transformation matrix *T*_I_^U^ between the ultrasound probe frame (*O*_U_) and the ultrasound image frame (*O*_I_). A mountain-type calibration template with three plates is fixed at the bottom of the water tank while a DRF (*O*_D_) is also mounted on the upper corner of the water tank. The position (*P*_D_) of the target point *P* relative to the tank DRF frame (*O*_D_) is calibrated prior to the experiment. A DRF (*O*_U_) is also attached on the ultrasound probe for position tracking of the probe. The calibration template is scanned by the ultrasound probe, and the image coordinate (*P*_I_) of the target point *P* is determined from the ultrasound image. The position of the target point *P* relative to the optical tracker frame can be expressed through either the tank DRF frame or the ultrasound probe frame as shown in

(1) $$\begin{array}{c} T_{D}^{T}P_{D}=T_{U}^{T}T_{I}^{U}P_{I}, \end{array}$$

where I represents the ultrasound image frame; U represents the ultrasound probe frame; T represents the optical tracker frame; D represents the tank DRF frame; *T*_D_^T^, *T*_U_^T^, *P*_D_, and *P*_I_ are known.

The transformation matrix *T*_I_^U^ can be determined by bringing the tracker and image coordinates of the target point *P* at three or more positions, *P*_*i*_ (*P*_D*i*_, *P*_I*i*_), *i* = 1, 2,…, *N*, *N* ≥ 3, into (1) and solved by optimization method such as the least square algorithm. After the transformation matrix *T*_I_^U^ has been determined, the coordinates of any target tumor detected by the ultrasound probe can be transferred and expressed relative to the optical tracker frame as described by

(2) $$\begin{array}{c} P_{T}=T_{U}^{T}\;T_{I}^{U}\;P_{I}. \end{array}$$

**Figure 5:**
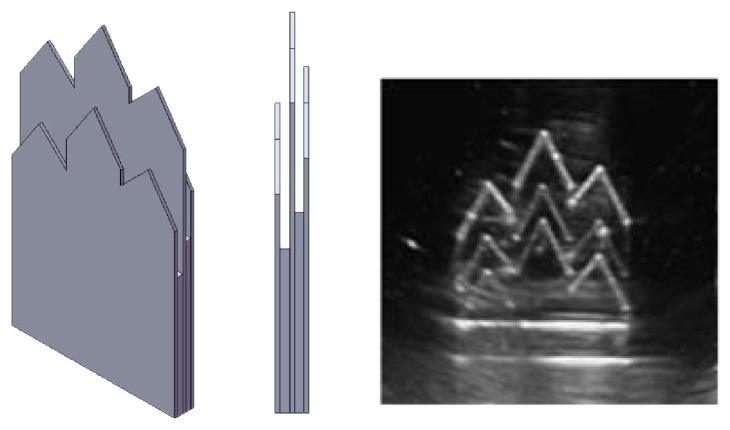
The calibration template and the boundary image of the middle plate, which is clearer than those of the other two plates.

The calibration template used for the registration of the optical tracker frame and the ultrasound image frame is shown in Figure 5. Since ultrasound scan beam has a slice thickness (elevational direction), it is necessary to determine the middle plane of the slice so that the following positioning calibration will be more precise. Therefore, a three-layer template is designed to make sure that the ultrasound scan is correctly located on the middle plate which will have brighter or clear boundary images than those of the other two plates.

### 2.3. Coordinate Transformation between the Robotic Arm and the Optical Tracker

**Figure 6:**
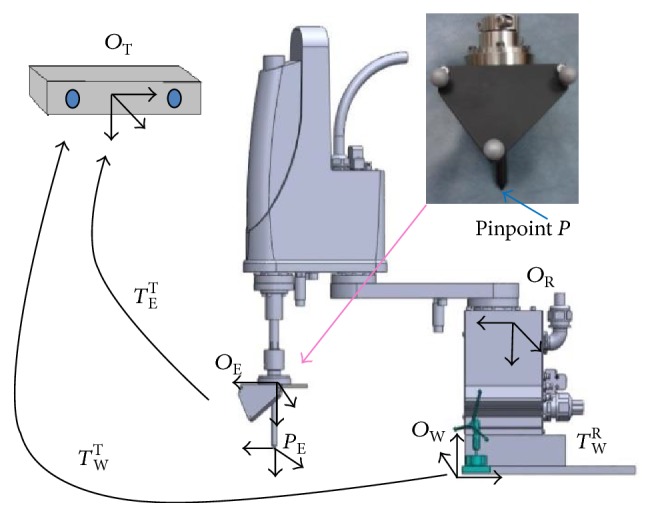
The coordinate transformation between the optical tracker and the robotic arm.

Figure 6 shows the coordinate transformation relationship between the optical tracker and the robotic arm. A tracking device mounted with a DRF (coordinate frame E) and a pin of 10 cm in length (pinpoint *P* represents the focal point of the HIFU transducer) is designed and mounted at the end effector of the robotic arm. A DRF is fixed on the robot base and used to define the world coordinate frame W in case the optical tracker is moved during the registration. The robot coordinate frame is defined as frame R. The transformation matrices *T*_W_^T^ and *T*_E_^T^ can be determined directly by the optical tracker. The transformation matrix *T*_W_^R^ will be solved so that the coordinates of the optical tracker frame can be transformed to the robot frame. In other words, according to the mathematical relationship we got from solving those matrices, the coordinates of any target point detected by the ultrasound probe can be transformed to robot frame through the optical tracker. *O*_E_ represents the position of the origin of the coordinate frame E. And (3) shows the relationship between *O*_E_ and *O*_W_ (the coordinate frame W).

(3) $$\begin{array}{c} O_{W}={\left(T_{W}^{T}\right)}^{-1}T_{E}^{T}O_{E}. \end{array}$$

If the robotic arm is manipulated to move around, the coordinates of point *O*_E_ relative to the coordinate frames R and W are calculated by the robotic arm controller and (3), respectively. Therefore, the transformation matrix *T*_W_^R^ between the robotic arm and world coordinate frame W can be determined by

(4) $$\begin{array}{c} O_{R}=T_{W}^{R}O_{W}. \end{array}$$

Because both *O*_W_ and *O*_R_ are not square matrices, we use least mean square algorithm to solve *T*_W_^R^.

(5) $$\begin{array}{c} T_{W}^{R}=O_{R}{O_{W}}^{T}{\left(O_{W}{O_{W}}^{T}\right)}^{-1}. \end{array}$$

**Figure 7:**
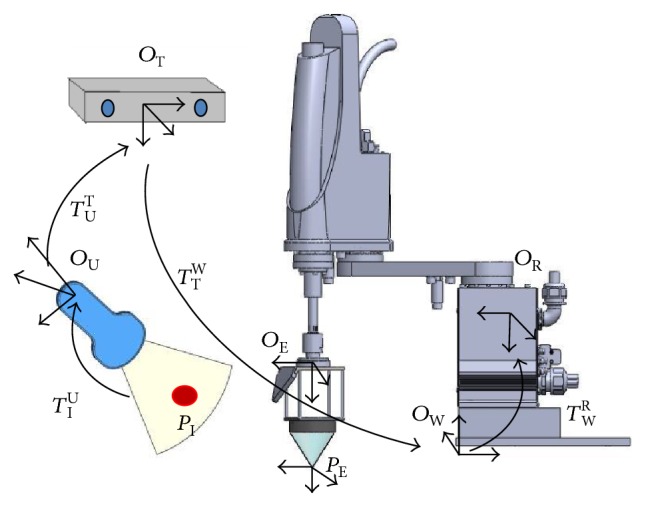
The coordinate transformation between the tumor *P*_I_ and the robotic arm.

Finally, after completing the registrations between the ultrasound image and the optical tracker as well as between the optical tracker and the robotic arm, the coordinates of the target tumor scanned and detected by the ultrasound system can be transformed and represented by the robot frame. The transformation relationship is defined by (6).  Figure 7 illustrated the whole coordinate transformation method.

(6) $$\begin{array}{c} P_{R}=T_{W}^{R}{T_{T}^{W}T}_{U}^{T}T_{I}^{U}P_{I}, \end{array}$$

where *P*_I_ is the image coordinate of the target tumor.

Figure 7 also shows that the HIFU transducer has been mounted to the end effector of the robotic arm for HIFU thermal treatment.

## 3. Experiment Results and Discussions

After building the whole ultrasound imaging-guided robotic HIFU system and finishing the preliminary works, we conducted three experiments as follows. The first one is the accuracy experiment to evaluate the coordination transformation accuracy between the ultrasound image and optical tracker. The second one is also the accuracy experiment to evaluate the coordination transformation accuracy between the ultrasound image and the robotic arm. The third experiment is to test the positioning accuracy of the entire system with ablating a phantom.

### 3.1. Accuracy Evaluation I: The Positioning Accuracy of Target Points

An experiment has been conducted to verify the positioning measurement error of the coordinate transformation between the ultrasound image and the optical tracker frame. The mountain-type template was seated in depth of 3 cm, 7 cm, and 12 cm. The template in each depth was scanned three times by the ultrasound probe. The distance error is defined as the difference between the coordinate of the target point under the ultrasound image coordinate system (*P*_I_) and the coordinate of the target point under the optical tracker coordinate system (*P*_T_). The distance errors of the three peak points (of the mountain-type template) in depth of 3 cm, 7 cm, and 12 cm are 0.67 ± 0.27 mm, 1.02 ± 0.26 mm, and 1.24 ± 0.24 mm, respectively.  Table 1 listed the experiment data of the cases in 3 cm, 7 cm, and 12 cm depth.

### 3.2. Accuracy Evaluation II: The Positioning Accuracy of the Robotic Arm

**Figure 8:**
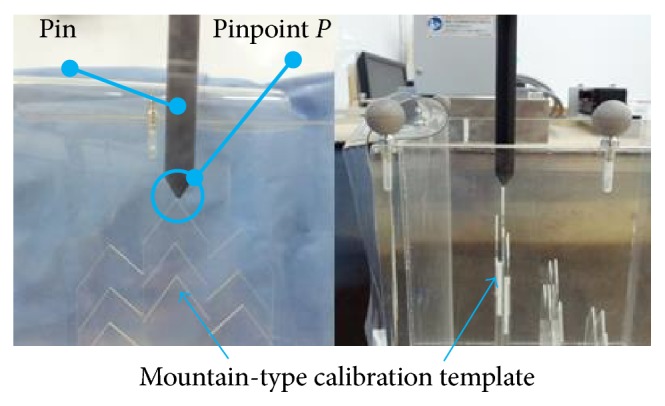
The pinpoint of the rod positions to the peak point of the calibration template.

The robotic arm was commanded to move to ten positions in order to calculate the transformation matrix *T*_W_^R^  by (5). After that, the calibration template was also seated in depth of 3 cm, 7 cm, and 12 cm and scanned by the ultrasound probe. There is a pin mounted on the end effector of the robotic arm (Figure 8). Then the robotic arm was commanded to move pinpoint *P* (the end of the pin) to the three peak points of the template (as shown in Figure 8). The distance errors between the peak points and the pinpoint *P* are listed in Table 2. The distance errors in depth of 3 cm, 7 cm, and 12 cm are 0.72 ± 0.26 mm, 1.02 ± 0.26 mm, and 1.31 ± 0.23 mm, respectively.

### 3.3. Accuracy Evaluation III: The Positioning Accuracy of the Ultrasound Imaging-Guided Robotic HIFU System with Ablating a Phantom

**Figure 9:**
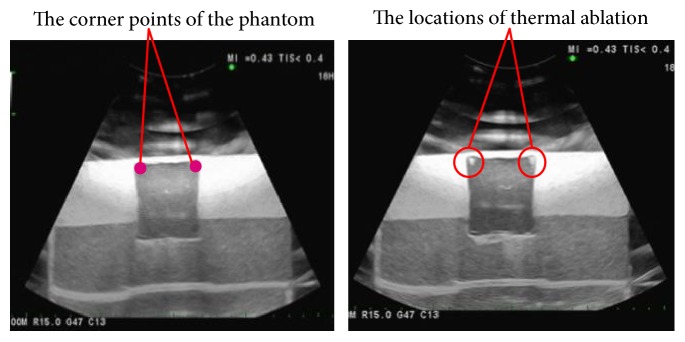
Positioning experiment of the HIFU thermal ablation.

The ultrasound imaging-guided robotic HIFU treatment experiment was conducted by commanding the robotic arm to move the HIFU focal point to ablate the four corner points of a phantom, which was detected by ultrasound images.  Figure 9 shows that the HIFU focal point can be positioned to the target (corner) points for thermal ablation. The average distance error is 1.32 ± 0.58 mm, and the distance error of each corner point is listed in Table 3.

## 4. Conclusions

This study proposes an ultrasound imaging-guided robotic HIFU experimental system for thermal ablation of tumors. By using this system, the positioning coordinates of targets (which are determined by the ultrasound imaging system) are transformed to the robot coordinate frames so that the robotic arm can move the HIFU transducer to ablate the target tumors. Instead of building the huge, solid, and costly system, this system tries to combine with the existing ultrasound imaging equipment to achieve HIFU ablation function.

The positioning accuracy evaluation results in Section 3 show that the distance error of the ultrasound imaging-guided robotic HIFU system is 1.32 ± 0.58 mm. However, for clinical use, this system still has many things needed to improve. So far, this study has built an experimental HIFU treatment system and confirmed its possibility and accuracy. The next step of this research is to consider the path planning issue and the respiration problem (respiration might cause tumor moving during the HIFU treatment [14]) in order to get more closer to deal with a real HIFU treatment situation.

## Figures and Tables

**Table 1:** The distance error of three peak points (of the calibration template) (unit: mm).

Number of point	Coordinates of the target point			Number of image	Coordinates of the guided pinpoint			Distance error
	*x*	*y*	*z*		*x*	*y*	*z*	
Case A: 3 cm								
1	26.7	8.9	−46.5	I	26.6	8.6	−46.8	0.44
				II	27.4	8.4	−46.4	0.87
				III	27.1	8.4	−46.7	0.70

2	6.2	−5.4	−45.2	I	5.3	−4.5	−44.9	1.31
				II	6.0	−5.2	−44.8	0.49
				III	6.3	−5.0	−44.6	0.73

3	1.8	−19.1	−61.5	I	2.0	−19.3	−62.0	0.57
				II	1.9	−19.6	−61.9	0.65
				III	1.8	−19.4	−61.6	0.31

Max error: 1.31; average error: 0.67; standard deviation 0.27								

Case B: 7 cm								
1	38.9	27.9	−89.6	I	39.2	28.1	−90.5	0.97
				II	39.1	28.0	−90.7	1.12
				III	39.6	27.8	−90.5	1.14

2	18.3	14.0	−87.7	I	17.5	13.8	−88.5	1.15
				II	18.0	13.6	−88.1	0.64
				III	18.1	13.9	−88.2	0.55

3	13.7	−0.1	−104.5	I	14.1	−0.5	−105.4	1.06
				II	14.0	0.0	−105.9	1.43
				III	14.2	−0.2	−105.5	1.12

Max error: 1.43; average error 1.02; standard deviation: 0.26								

Case C: 12 cm								
1	54.1	46.8	−136.3	I	55.4	46.7	−136.2	1.31
				II	53.5	47.5	−137.6	1.59
				III	53.9	47.3	−137.3	1.14

2	33.2	32.9	−134.6	I	34.5	32.8	−134.2	1.36
				II	32.7	33.4	−135.3	0.99
				III	32.8	32.9	−135.4	0.89

3	28.9	19.0	−151.6	I	29.8	19.4	−151.4	1.00
				II	28.6	20.1	−152.1	1.24
				III	28.0	19.2	−152.9	1.59

Max error: 1.59; average error: 1.24; standard deviation: 0.24								

**Table 2:** The distance error of the robotic arm (unit: mm).

Number of point	Coordinates of the target point			Number of image	Coordinates of the guided pinpoint			Distance error
	*x*	*y*	*z*		*x*	*y*	*z*	
Case A: 3 cm								
1	26.7	8.9	−46.5	I	27.0	8.2	−46.6	0.78
				II	27.4	8.1	−46.5	1.06
				III	26.5	8.4	−46.7	0.57

2	6.2	−5.4	−45.2	I	6.1	−5.3	−44.9	0.33
				II	6.3	−5.2	−44.6	0.64
				III	5.3	−4.6	−45.1	1.20

3	1.8	−19.1	−61.5	I	1.9	−19.3	−62.1	0.64
				II	1.9	−19.6	−62.1	0.78
				III	1.7	−19.5	−61.8	0.50

Max error: 1.20; average error: 0.72; standard deviation: 0.26								

Case B: 7 cm								
1	38.9	27.9	−89.6	I	39.2	27.8	−90.5	0.95
				II	39.2	27.9	−90.6	1.04
				III	39.6	27.8	−90.4	1.06

2	18.3	14.0	−87.7	I	18.1	13.5	−88.2	0.73
				II	17.5	13.7	−88.5	1.17
				III	18.2	13.8	−88.1	0.45

3	13.7	−0.1	−104.5	I	14.0	−0.6	−105.5	1.15
				II	14.2	−0.4	−105.6	1.24
				III	14.1	−0.1	−105.8	1.36

Max error: 1.36; average error: 1.02; standard deviation: 0.26								

Case C: 12 cm								
1	54.1	46.8	−136.3	I	55.4	46.6	−136.1	1.33
				II	53.5	47.4	−137.6	1.55
				III	53.9	47.2	−137.4	1.19

2	33.2	32.9	−134.6	I	34.5	32.7	−134.2	1.37
				II	32.6	33.4	−135.3	1.05
				III	32.8	33.6	−135.3	1.07

3	28.9	19.0	−151.6	I	29.8	19.4	−151.3	1.03
				II	28.0	20.1	−152.1	1.51
				III	28.0	19.3	−153.0	1.69

Max error: 1.69; average error: 1.31; standard deviation: 0.23								

**Table 3:** The distance error of HIFU thermal ablation (unit: mm).

Number of points	Position of the target (mm)			Position of the ablation (mm)			Distance error
	*x*	*y*	*z*	*x*	*y*	*z*	
1	−71.6	248.6	79.7	−71.4	248.2	80.0	0.53
2	−70.6	289.2	81.2	−72.6	288.6	81.1	2.09
3	−112.0	248.4	79.63	−111.0	248.1	79.7	1.05
4	−109.0	289.5	81.24	−108.0	288.7	80.3	1.59
